# Laparoscopic extended right hemicolectomy with D3 lymph node dissection using a new articulating instrument

**DOI:** 10.1007/s10151-020-02345-z

**Published:** 2020-09-14

**Authors:** H. Y. Jin, C. S. Lee, Y. S. Lee

**Affiliations:** Division of Colorectal Surgery, Department of Surgery, College of Medicine, Seoul St. Mary’s Hospital, The Catholic University of Korea, 222, Banpo-daero, Seocho-gu, Seoul, 06591 Republic of Korea

In this video, we show the use of an articulating laparoscopic instrument (ArtiSential®, LIVSMED, Inc., Republic of Korea) registered as a class I medical device with the Food and Drug Administration in 2019 and available since November 2019 in Korea (Fig. 1).

**Fig. 1:**
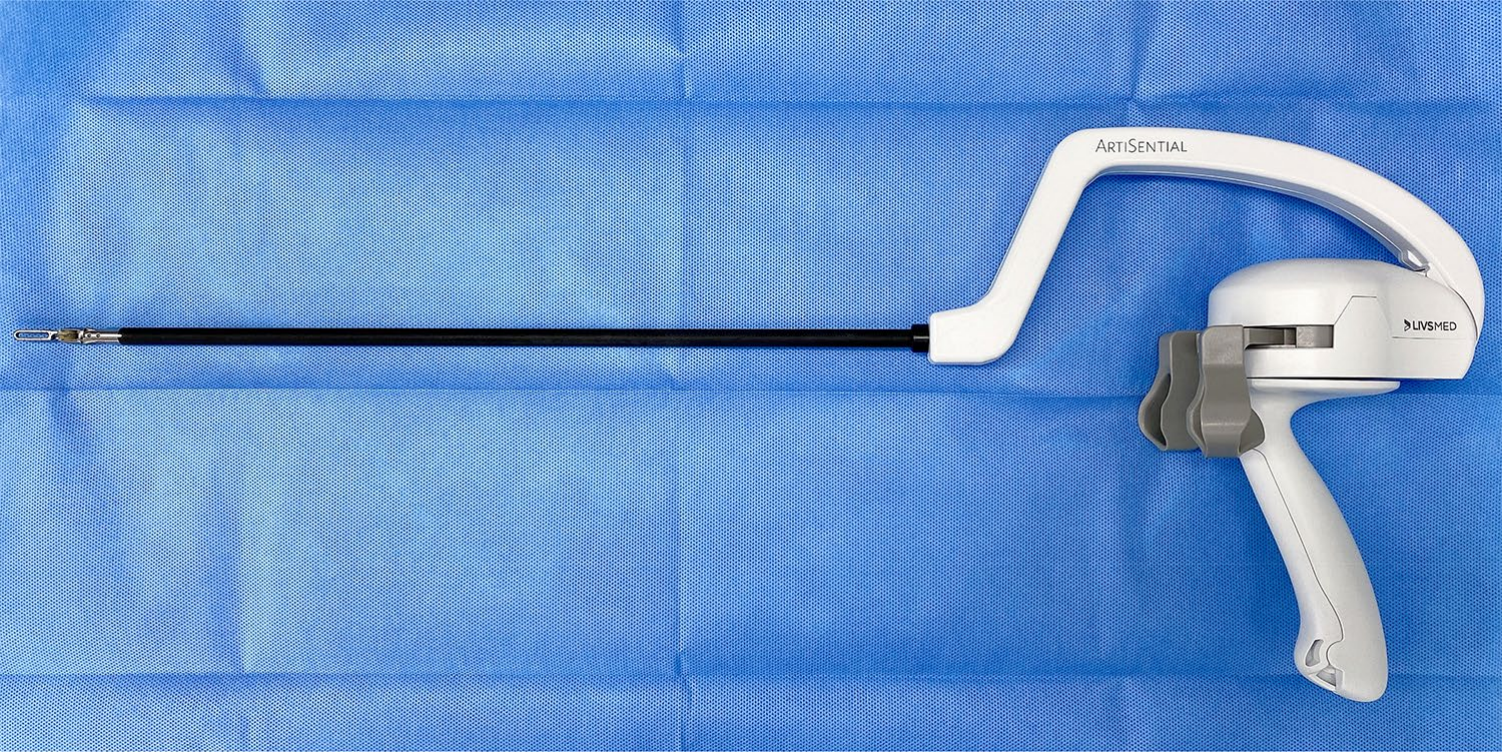
The laparoscopic articulating instrument ArtiSential®

Since the concept of complete mesocolic excision (CME) was introduced by Hoenberger et al. [1], CME with D3 lymph node dissection has been considered to improve oncologic outcomes. However, laparoscopic right hemicolectomy, including CME with D3 lymph node dissection, is technically challenging, particularly in ligation of the major vessels at their origin sites and lymphadenectomy along the superior mesenteric axis [2]. Obtaining the effective angle, traction, and countertraction is difficult using conventional straight-fixed laparoscopic instruments [3]. To overcome these limitations, a surgical robot system was introduced for colorectal surgery. Robot systems have the advantages of multi-joint instruments, ergonomics, and three-dimensional vision but are still expensive. As an alternative to robot systems, several laparoscopic articulating instruments have been introduced but are unfortunately not of practical use. In this video, we present laparoscopic extended right hemicolectomy using a new laparoscopic articulating instrument.

The patient in the video is a 72-year-old woman with a body mass index of 23.3 kg/m^2^ at the time of surgery. She was diagnosed with a well-differentiated adenocarcinoma at the hepatic flexure of the colon. Initial computed tomography (CT) scan revealed clinical stage T3N1 and no evidence of distant metastasis. We performed laparoscopic extended right hemicolectomy with D3 lymph node dissection.

The patient was placed in a modified lithotomy position with both arms along the body. A camera port was placed at the umbilicus, and three 5-mm ports were placed in the right lower quadrant, right upper quadrant, and left upper quadrant. An 8-mm port was placed in the left lower quadrant (Fig. 2). During the procedures, the surgeon used the ArtiSential® instrument through the left lower quadrant port and advanced surgical energy device (HARMONIC HD1000i, Ethicon, Cincinnati, OH, USA) through the left upper quadrant port (see video). Mobilization of the mesocolon was started from the ileocecal junction and dissected through the avascular plane between the mesocolon and the retroperitoneum using a monopolar electrosurgery hook. The Toldt’s fascia, duodenum, and right ureter were preserved during the dissection. The ileocolic pedicle was identified by gentle traction using an articulation instrument, and ileocolic vessels were dissected and ligated at their origin sites. Subsequently, the middle colic pedicle was identified by gentle traction using an articulation instrument, and middle colic artery was ligated at its origin site [4]. The right colon and hepatic flexure were mobilized, the umbilical port was extended, and the specimen was extracted. Extracorporeal end-to-side anastomosis was performed after checking the perfusion status using indocyanine green.

**Fig. 2:**
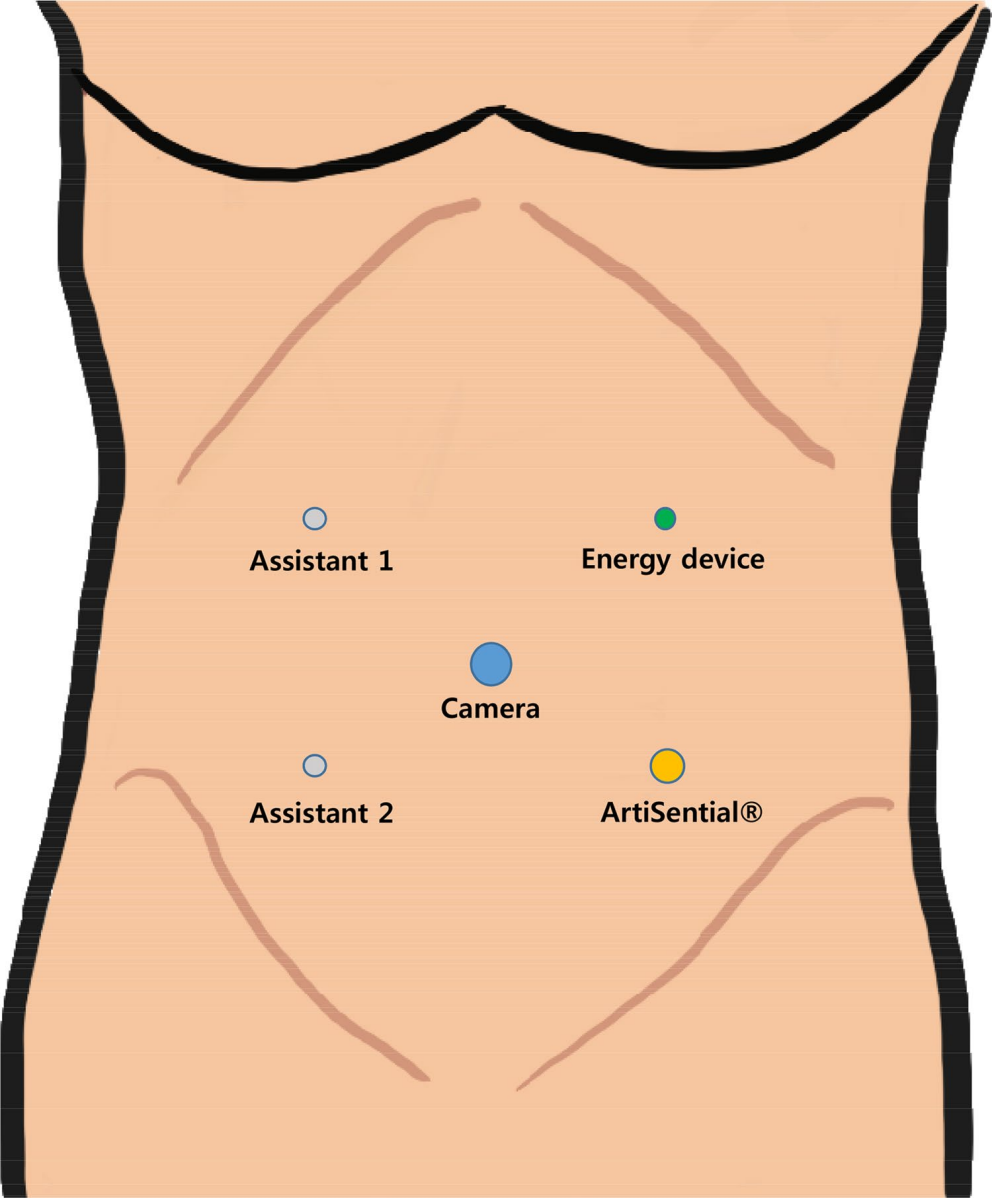
Port placement. An 8-mm port was placed in the left lower quadrant using the ArtiSential® instrument

The total operation time was 165 min, with an estimated blood loss of 20 mL. There were no intra- or postoperative complications, and the patient was discharged on postoperative day 3. The final pathology was pT3N1bM0. Forty-six regional lymph nodes were harvested, and two metastatic regional lymph nodes were identified.

The new laparoscopic articulating instrument (ArtiSential®, LIVSMED, Inc., Republic of Korea) helps surgeons to obtain the effective traction and countertraction easily through its intuitive movement. This instrument has a vertical and horizontal joint structure that is synchronized with the user’s hands and provides angles of 360°. Its multiple degrees of movement allow a wider variety of surgical procedures than those with straight-fixed laparoscopic instruments.

This is the first video presenting the clinical application of a newly launched, articulating laparoscopic instrument (ArtiSential®, LIVSMED, Inc., Republic of Korea). In this video, laparoscopic extended right hemicolectomy using an articulating laparoscopic instrument is safe and technically feasible. This instrument is ergonomic, allows intuitive surgery, and is less expensive than a robot system. Future comparative studies with conventional laparoscopic or robotic surgery are required to establish its benefits for clinical applications.

## Electronic supplementary material

Below is the link to the electronic supplementary material.

Supplementary file1 (MP4 449691 kb)
